# Enhanced photoelectrochemical water splitting performance of α-Fe_2_O_3_ photoanodes through Co-modification with Co single atoms and g-C_3_N_4_†

**DOI:** 10.1039/d4sc03442b

**Published:** 2024-07-05

**Authors:** Juan Wu, Xiaodi Du, Mingjie Li, Hongyu Chen, Bin Hu, Hongwei Ding, Nannan Wang, Lin Jin, Weisheng Liu

**Affiliations:** a Henan Key Laboratory of Rare Earth Functional Materials, International Joint Research Laboratory for Biomedical Nanomaterials of Henan, Zhoukou Normal University Zhoukou 466001 P. R. China jinlin_1982@126.com; b College of Chemistry and Chemical Engineering, Zhoukou Normal University Zhoukou 466001 P. R. China; c Library, Zhoukou Normal University Zhoukou 466001 P. R. China; d Key Laboratory of Nonferrous Metal Chemistry and Resources Utilization of Gansu Province, State Key Laboratory of Applied Organic Chemistry, College of Chemistry and Chemical Engineering, Lanzhou University Lanzhou 730000 P. R. China liuws@lzu.edu.cn

## Abstract

The practical application of α-Fe_2_O_3_ in water splitting is hindered by significant charge recombination and slow water oxidation. To address this issue, a CoSAs–g-C_3_N_4_/Fe_2_O_3_ (CoSAs: cobalt single atoms) photoanode was fabricated in this study through the co-modification of CoSAs and g-C_3_N_4_ to enhance photoelectrochemical (PEC) water splitting. The coupling between g-C_3_N_4_ and α-Fe_2_O_3_ resulted in the formation of a heterojunction, which provided a strong built-in electric field and an additional driving force to mitigate charge recombination. Moreover, g-C_3_N_4_ served as a suitable carrier for single atoms, which effectively anchored CoSAs through N/C coordination. The highly dispersed CoSAs provided abundant active sites, which further promoted surface holes extraction and oxidation kinetics, resulting in higher PEC performance and photostability. This study indicates the benefits of these collaborative strategies and provides more efficient designs for solar energy conversion in PEC systems.

## Abstract 2

In this study, a photoanode material, g-C_3_N_4_/Fe_2_O_3_ anchored with Co single atoms (Co SAs) (CoSAs-g-C_3_N_4_/Fe_2_O_3_), was designed and assembled to improve water oxidation performance. The synergistic effect of g-C_3_N_4_/Fe_2_O_3_ heterojunction and Co SAs facilitated efficient charge separation.

## Graphical abstract

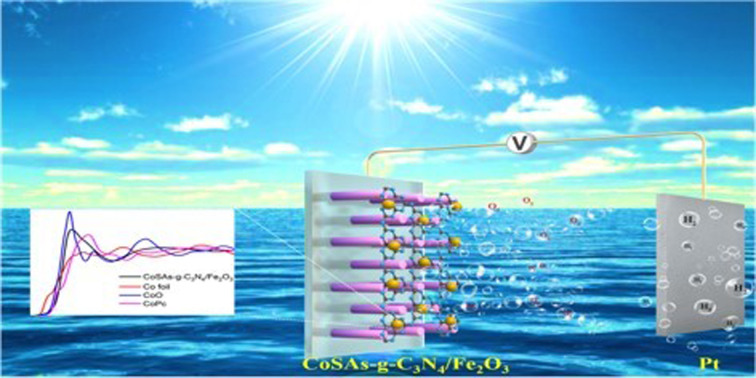

## Introduction

Photoelectrochemical (PEC) water splitting technology converts renewable solar energy into storable chemical energy. Moreover, this technology is considered a promising strategy for addressing energy crises and mitigating environmental pollution.^1–4^ Hematite (α-Fe_2_O_3_), a highly attractive photoanode material, has received significant attention in PEC water splitting owing to its narrow band gap (1.9–2.2 eV), which enables efficient absorption of visible light. Additionally, α-Fe_2_O_3_ exhibits high theoretical solar-to-hydrogen conversion efficiency (∼15.5%), earth-abundance, and stability.^5–8^ However, the significant challenge in charge recombination hinders the practical application of α-Fe_2_O_3_.^9^ Currently, the reported current density of α-Fe_2_O_3_ is significantly below its theoretical value (12.4 mA cm^−2^), indicating significant potential for improvement.^10^ Therefore, it is crucial and challenging to further modify α-Fe_2_O_3_ to improve its efficiency in separating photogenerated carriers.

In recent years, single atom catalysts (SACs) have emerged as a promising area of study owing to their various advantages,^11^ such as: (1) single metal atoms often serve as unsaturated coordination sites and are considered active centers; (2) strong metal–support interactions can increase the electron transfer between them, thereby regulating the electronic structure of metal atoms; (3) SACs can optimize the exposure of active sites and improve atomic utilization, leading to reduced catalytic costs. Owing to these advantages, SACs exhibit excellent activity and durability in numerous catalytic reactions, making them highly promising for applications in water splitting. For example, Bi *et al.* demonstrated the charge transfer and bond evolution between single atom Pt and C_3_N_4_ catalysts in photocatalytic water splitting.^12^ Lee *et al.* reported the CoSAs–MoS_2_/TiN nanorod (NR) electrocatalysts (CoSAs: cobalt single atoms). To the strong interaction between CoSAs and MoS_2_, adjusted their electron density distribution, resulting in more catalytic active sites for reactant molecules. This contributed to the excellent overall hydrolysis performance of the electrocatalyst in pH universal electrolytes.^13^ Additionally, Corvini *et al.* reported that single atom Ru anchored to NiFe-layered double hydroxides significantly enhanced the oxygen evolution activity of BiVO_4_.^14^ Transition metal Co exhibits unique physicochemical properties, making it a promising candidate material for SACs. Previous research has shown that Co SACs exhibit excellent electrocatalytic activity compared with Co, Fe, Ni, Cr, V, and Rh SACs.^15^ Considering that Co SACs have demonstrated effectiveness in improving catalytic activity,^16^ we suggest that incorporating (CoSAs) into α-Fe_2_O_3_ can further improve the PEC water oxidation performance by regulating charge distribution and increasing the reaction site. However, reports on CoSAs/Fe_2_O_3_ are scarce.^17^

Despite the excellent performance of SACs, the selection of a suitable carrier is crucial. Owing to the tendency of individual atoms to readily aggregate, only appropriate charge carriers can effectively and stably disperse these atoms. Currently, reports on CoSAs/Fe_2_O_3_ photoanodes are few, partly owing to the inadequacy of α-Fe_2_O_3_ as a suitable carrier. Therefore, introducing an additional suitable carrier medium into the photoanode system is crucial. The conjugated polymer graphite carbon nitride (g-C_3_N_4_) is considered a suitable candidate for a single atom carrier. g-C_3_N_4_ contains electron-rich N atoms, which can provide sufficient coordination sites and effectively anchor isolated metal atoms to nitrogen coordination centers, forming an M–N_*x*_ structure that serves as an active center for several catalytic reactions.^11,12,18^ For example, Zhu *et al.* synthesized Co–g-C_3_N_4_/rGO SACs. Owing to the formation of Co–N and Co–3N coordination structures, Co–g-C_3_N_4_/rGO SACs exhibit higher hydrogen evolution activity compared with commercial Pt/C. Moreover, these compounds exhibit stability for up to 500 h at high temperatures.^15^ Additionally, g-C_3_N_4_, a non-metallic semiconductor, can form heterojunctions with other semiconductors, thereby facilitating charge migration and separation owing to its suitable band structure. This characteristic has broad potential applications in photocatalysis. For example, Huang *et al.* investigated the g-C_3_N_4_/Mn_2_O_3_/FTO p–n heterojunction as a photoelectrode for PEC water decomposition.^19^ Similarly, Fu *et al.* synthesized an Au/g-C_3_N_4_/TiO_2_ nanotube array heterojunction as a photocatalyst for degrading *o*-chloronitrobenzene target pollutants.^20^ These studies indicated that g-C_3_N_4_ has the potential to serve as a carrier for CoSAs and a semiconductor to form heterojunctions with α-Fe_2_O_3_, playing a mutually beneficial role.

Herein, a photoanode material, g-C_3_N_4_/Fe_2_O_3_ anchored with CoSAs (CoSAs–g-C_3_N_4_/Fe_2_O_3_), was designed and assembled to improve water oxidation performance. Scanning transmission electron microscopy (STEM) and X-ray absorption fine structure analyses indicated that Co atoms were securely anchored on g-C_3_N_4_ through N/C coordination bonds, thereby maintaining atomic isolation. The electrochemical results revealed that the strong built-in electric field and photovoltage generated at the g-C_3_N_4_/Fe_2_O_3_ interface effectively reduced the recombination of photogenerated charges. Furthermore, the dispersed CoSAs provided sufficient active sites for water oxidation reactions. The synergistic effect of these favorable factors facilitated efficient charge separation. The optimized CoSAs–g-C_3_N_4_/Fe_2_O_3_ photoanode exhibited a photocurrent density of 1.93 mA cm^−2^ at 1.23 V_RHE_, which was 3.22 times that of pure α-Fe_2_O_3_. The potential charge transfer pathways and PEC water oxidation mechanism of CoSAs–g-C_3_N_4_/Fe_2_O_3_ were extensively elucidated.

## Experimental section

### Synthesis of the α-Fe_2_O_3_ nanorod array (NRA) photoanode

The α-Fe_2_O_3_ NRA sample was prepared using our previously reported method.^21,22^ Initially, 10 mL of aqueous solution containing 1.5 mmol FeCl_3_·6H_2_O and 1.5 mmol urea was transferred to a Teflon-lined stainless steel autoclave. Subsequently, a piece of cleaned FTO was placed in the aqueous solution. The autoclave was sealed and heated in an oven at 100 °C for 11 h. After the completion of the reaction and cooling to room temperature, the FTO substrate with the FeOOH film was removed, washed, and dried. The FeOOH film was then calcined in a muffle furnace at 550 °C for 2 h and at 700 °C for 10 min in an air atmosphere. Finally, the target α-Fe_2_O_3_ NRA grown on the FTO substrate was obtained and denoted as pure α-Fe_2_O_3_.

### Synthesis of the g-C_3_N_4_/Fe_2_O_3_ NRA photoanode

The preparation process of g-C_3_N_4_/Fe_2_O_3_ is as follows: first, an 10 mL ethylene glycol solution containing a certain amount of melamine was dripped onto the α-Fe_2_O_3_ NRA photoanode, followed by drying in a muffle furnace at 220 °C to eliminate the ethylene glycol from the surface of α-Fe_2_O_3_. This procedure was repeated twice to increase the deposition amount of melamine. Finally, α-Fe_2_O_3_ NRA coated with melamine were annealed at 550 °C for 2 h in a tube furnace in an Ar atmosphere to produce the g-C_3_N_4_/Fe_2_O_3_ NRA photoanode. The thickness of the g-C_3_N_4_ layer was optimized by adjusting the amount of melamine added to the glycol solution. The resulting g-C_3_N_4_/Fe_2_O_3_ was labeled as g-C_3_N_4_/Fe_2_O_3_-*x*, with *x* representing the amount of melamine added to the ethylene glycol solution (*e.g.*, g-C_3_N_4_/Fe_2_O_3_-0.1, g-C_3_N_4_/Fe_2_O_3_-0.2, and g-C_3_N_4_/Fe_2_O_3_-0.3 for 0.1, 0.2, and 0.3 g, respectively). The photoelectrochemical performance test revealed that g-C_3_N_4_/Fe_2_O_3_-0.2 exhibited the highest performance (Fig. S1†). Therefore, g-C_3_N_4_/Fe_2_O_3_ in the study denotes g-C_3_N_4_/Fe_2_O_3_-0.2, unless stated otherwise.

### Synthesis of the CoSAs–g-C_3_N_4_/Fe_2_O_3_ NRA photoanode

CoSAs were anchored on the g-C_3_N_4_/Fe_2_O_3_ NRA surface as follows: initially, 0.88 g of Co(NO_3_)_2_·6H_2_O was dissolved in 100 mL of deionized water and stirred for 30 min. Subsequently, g-C_3_N_4_/Fe_2_O_3_ NRA were immersed in the above solution and maintained at 50 °C for 5 h. Afterward, g-C_3_N_4_/Fe_2_O_3_ NRA were removed, washed several times with ethanol and deionized water, and dried at 60 °C. Finally, the sample was annealed in an Ar atmosphere at 400 °C for 2 h with a heating rate of 2 °C min^−1^ to synthesize CoSAs–g-C_3_N_4_/Fe_2_O_3_ photoanode.

## Results and discussion

**Fig. 1:**
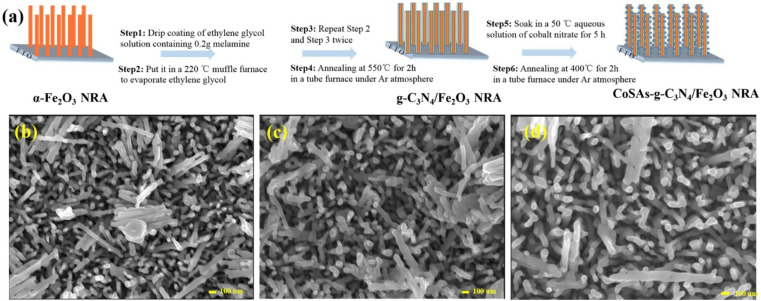
(a) Schematic of the CoSAs–g-C_3_N_4_/Fe_2_O_3_ NRA photoanode preparation process. Top-view SEM images of (b) α-Fe_2_O_3_; (c) g-C_3_N_4_/Fe_2_O_3_; (d) CoSAs–g-C_3_N_4_/Fe_2_O_3_.

Fig. 1a illustrates the schematic of the preparation process for the CoSAs–g-C_3_N_4_/Fe_2_O_3_ photoanode. Initially, melamine was uniformly deposited on the surface of α-Fe_2_O_3_ by dripping a glycol solution containing melamine.^20^ Subsequently, α-Fe_2_O_3_ coated with melamine was thermally condensed in a tube furnace to produce the g-C_3_N_4_/Fe_2_O_3_ composite. Unlike g-C_3_N_4_/Fe_2_O_3_ formed through the spin-coating of g-C_3_N_4_ on the α-Fe_2_O_3_ surface, g-C_3_N_4_ produced during the thermal condensation process can form a strong chemical bond with α-Fe_2_O_3_. This prevented defects resulting from the mismatched layers in the heterostructure caused by the direct spin-coating of g-C_3_N_4_ on the α-Fe_2_O_3_ surface. These defects often served as sites for the undesirable recombination of photogenerated carriers. Moreover, the use of g-C_3_N_4_ as the substrate facilitated the anchoring of CoSAs onto g-C_3_N_4_/Fe_2_O_3_ through water bath deposition and calcination methods, resulting in the target CoSAs–g-C_3_N_4_/Fe_2_O_3_ NRA photoanode. Scanning electron microscopy (SEM) images (Fig. 1b–d) revealed that pure α-Fe_2_O_3_ exhibited a nanorod array structure, vertically grown on the FTO substrate. Conversely, the morphologies of g-C_3_N_4_/Fe_2_O_3_ and CoSAs–g-C_3_N_4_/Fe_2_O_3_ remained unchanged, indicating that g-C_3_N_4_ maintained an ultra-thin structure, and the dispersed CoSAs had no effect on the morphology of g-C_3_N_4_/Fe_2_O_3_. The X-ray diffraction (XRD) spectra of g-C_3_N_4_/Fe_2_O_3_ and CoSAs–g-C_3_N_4_/Fe_2_O_3_ (Fig. S2†) exhibited diffraction peaks corresponding to α-Fe_2_O_3_ (JCPDS No. 33-0664) and FTO.^5,23^ No characteristic peaks associated with g-C_3_N_4_ and Co components were observed, likely owing to the low amount and poor crystallinity of g-C_3_N_4_ and the trace amount of dispersed CoSAs. These Co atoms did not aggregate into Co nanoparticles, which are crucial for producing prominent diffraction peaks.

**Fig. 2:**
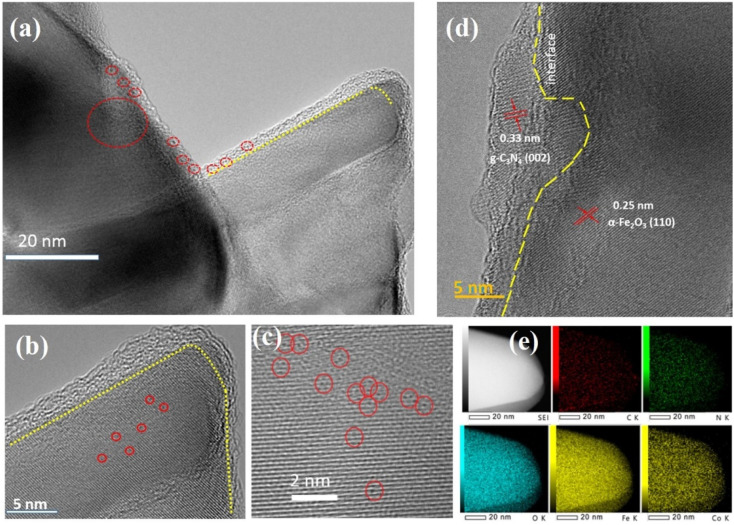
(a)–(c) Atomic-resolution TEM images of CoSAs–g-C_3_N_4_/Fe_2_O_3_ NR (CoSAs are highlighted by red circles); (d) and (e) HRTEM image and EDS mapping of Co, Fe, O, C, and N for CoSAs–g-C_3_N_4_/Fe_2_O_3_ NR.

To confirm the presence of g-C_3_N_4_ and CoSAs, the aberration-corrected annular bright-field STEM (AC-ABF-STEM) imaging was performed on CoSAs–g-C_3_N_4_/Fe_2_O_3_. The AC-ABF-STEM image (Fig. 2a) revealed an ultra-thin layer of g-C_3_N_4_ surrounding α-Fe_2_O_3_ NR, forming a close interface, suggesting the formation of a heterojunction between g-C_3_N_4_ and α-Fe_2_O_3_.^24,25^ Additionally, numerous black dots corresponding to CoSAs were observed on g-C_3_N_4_ (marked by red circles), indicating the uniform dispersion of CoSAs without the presence of nanoparticles or clusters. The magnified AC-ABF-STEM images (Fig. 2b and c) revealed the poor crystallinity of the g-C_3_N_4_ layer, with an average thickness of ∼3 nm. The size of a single black dot corresponding to an isolated Co site was 0.15 nm. The high-resolution TEM (HRTEM) image (Fig. 2d) indicated a distinct interface structure with two lattice stripes. Particularly, the lattice stripe of 0.25 nm corresponded to the (110) crystal plane of α-Fe_2_O_3_.^26–28^ Another lattice stripe, observed within a thin layer of poor crystallinity, exhibited a lattice spacing of 0.33 nm, corresponding to the (002) crystal plane of g-C_3_N_4_.^20^ This strongly confirms that the ultra-thin layer with poor crystallization was g-C_3_N_4_, thereby validating the successful preparation of g-C_3_N_4_/Fe_2_O_3_ heterojunction. Moreover, TEM-EDS elemental mapping revealed a uniform distribution of Fe, O, C, N, and Co elements on NR (Fig. 2e), indicating the presence of the g-C_3_N_4_ layer and CoSAs. This confirms the successful formation of CoSAs–g-C_3_N_4_/Fe_2_O_3_ composite materials.

**Fig. 3:**
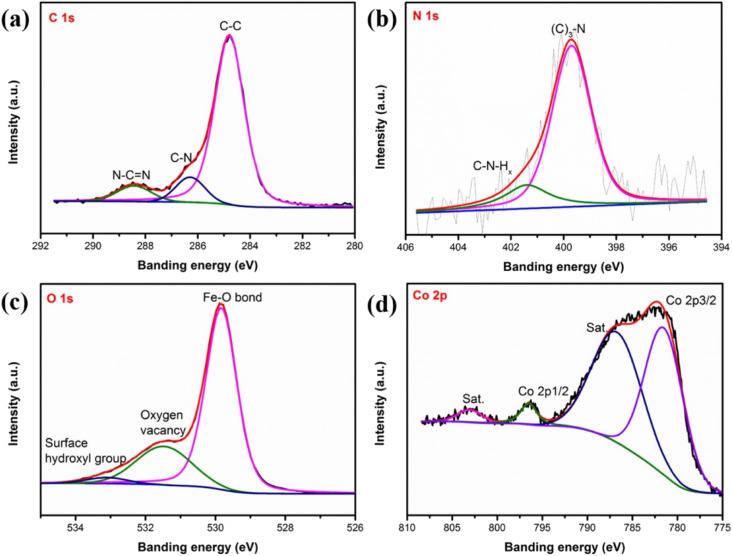
High-resolution XPS spectra of (a) C 1s; (b) N 1s; (c) O 1s; (d) Co 2p, for CoSAs–g-C_3_N_4_/Fe_2_O_3_.

To analyze the surface chemical states, we performed X-ray photoelectron spectroscopy (XPS) characterization on α-Fe_2_O_3_, g-C_3_N_4_/Fe_2_O_3_, and CoSAs–g-C_3_N_4_/Fe_2_O_3_. Fig. 3a–d show the XPS spectra of C 1s, N 1s, O 1s, and Co 2p, for CoSAs–g-C_3_N_4_/Fe_2_O_3_, respectively. The XPS spectra of C 1s (Fig. 3a) exhibited three peaks corresponding to C–C (284.8 eV), C–N (286.3 eV), and N–C=N (288.4 eV).^20^ The N 1s spectrum (Fig. 3b) featured two peaks at 399.7 and 401.4 eV, corresponding to N–(C)_3_ and C–NH_*x*_,^20^ respectively. These results from the C 1s and N 1s spectra indicate the successful loading of g-C_3_N_4_ on the α-Fe_2_O_3_ surface, resulting in the formation of a g-C_3_N_4_/Fe_2_O_3_ heterostructure. The O 1s spectrum (Fig. 3c) exhibited three peaks at around 529.8, 531.5, and 533.1 eV, which were assigned to the Fe–O bond,^10^ oxygen vacancies within the α-Fe_2_O_3_ structure,^29^ and surface hydroxyl groups, respectively.^30^ The Fe 2p spectrum (Fig. S3†) featured two peaks at 724.4 and 710.6 eV, corresponding to Fe 2p_1/2_ and Fe 2p_3/2_, respectively, along with two satellite peaks at 732.7 and 718.2 eV, consistent with the reported values for Fe^3+^ in Fe_2_O_3_.^31^ Additionally, the Fe 2p_3/2_ peak can be deconvoluted into three peaks at 712.2, 710.6, and 709.6 eV, indicating the characteristics of Fe^3+^. The Co 2p spectra (Fig. 3d) exhibited two main peaks corresponding to Co 2p_3/2_ (781.5 eV) and Co 2p_1/2_ (796.5 eV),^15^ along with satellite peaks, suggesting the successful anchoring of CoSAs on g-C_3_N_4_. Notably, after the loading of CoSAs and g-C_3_N_4_, the Fe 2p, O 1s, and N 1s peaks slightly shifted to higher binding energies, suggesting interactions between CoSAs, g-C_3_N_4_, and α-Fe_2_O_3_ (Fig. S4†).^14^

**Fig. 4:**
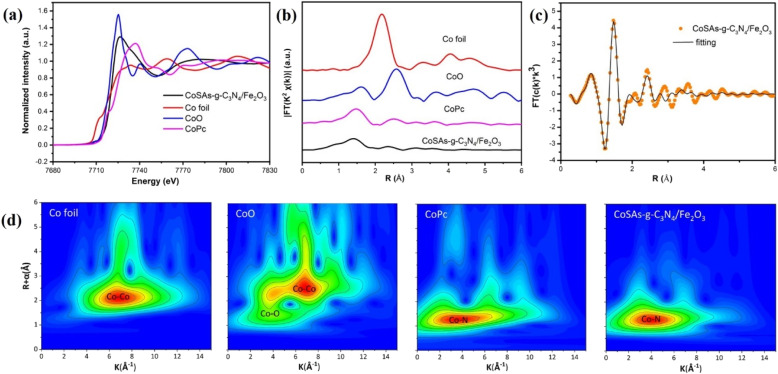
(a) XANES spectra; (b) *R*-space Co K-edge EXAFS spectra; (c) EXAFS *R*-space fitting curve; (d) WT-EXAFS signals of Co foil, CoO, CoPc and CoSAs–g-C_3_N_4_/Fe_2_O_3_.

Furthermore, X-ray absorption near-edge structure (XANES) analysis was performed to investigate the oxidation state and coordination structure of Co atoms in CoSAs–g-C_3_N_4_/Fe_2_O_3_. The XANES spectra of CoSAs–g-C_3_N_4_/Fe_2_O_3_ (Fig. 4a) indicated the unique oxidation state of Co^*δ*+^ (0 < *δ* < 2), which can be supported by the linear combination fitting (LCF) result (Fig. S5†). The extended X-ray absorption fine structure (EXAFS) spectra of CoSA–g-C_3_N_4_/Fe_2_O_3_ (Fig. 4b) exhibited a single dominant peak at 1.5 Å, corresponding to the first coordination shell of Co–N. Notably, the weaker peak intensity of the CoSAs–g-C_3_N_4_/Fe_2_O_3_ spectrum compared with CoPc suggested a lower Co–N coordination value and inferior crystal properties.^32^ Additionally, the absence of the characteristic Co–Co bond length (∼2.1 Å) in Co foils within CoSAs–g-C_3_N_4_/Fe_2_O_3_, indicated the lack of Co nanoparticles, consistent with atomic-resolution TEM data (Fig. 2a–c) and XRD pattern (Fig. S2†). The coordination of Co sites was further investigated through EXAFS curve fitting analysis (Fig. 4c), confirming that each Co atom was coordinated with 4.1 ± 0.2 N atoms at the first shell and 1.1 ± 0.4 C atoms at second shell according to the fitting results (Fig. S6 and Table S1†). Notably, unlike the reference samples, a strong peak of Co–N coordination in the wavelet transforms (WT) of Co K-edge EXAFS oscillations (Fig. 4d), directly confirmed the presence of CoSAs on the g-C_3_N_4_/Fe_2_O_3_ substrate. All results obtained from atomic-resolution STEM, HRTEM, XPS, and XANES confirmed the successful preparation of the CoSAs–g-C_3_N_4_/Fe_2_O_3_ photoanode.

**Fig. 5:**
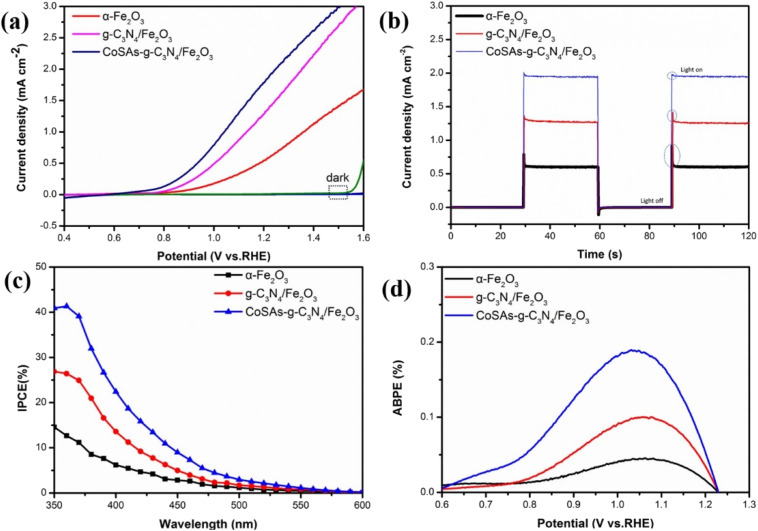
(a) LSV curves; (b) transient photocurrent measurements at 1.23 V_RHE_; (c) IPCE curves; (d) ABPE curves of α-Fe_2_O_3_, g-C_3_N_4_/Fe_2_O_3_, and CoSAs–g-C_3_N_4_/Fe_2_O_3_ photoanodes.

The PEC water oxidation properties of the obtained photoanodes were measured using a three-electrode system. The linear sweep voltammetry (LSV) curves (Fig. 5a) revealed that pure α-Fe_2_O_3_ exhibited a photocurrent density of 0.59 mA cm^−2^ at 1.23 V_RHE_ with an initial potential of 0.775 V_RHE_. Upon coupling α-Fe_2_O_3_ with g-C_3_N_4_, the photocurrent density of g-C_3_N_4_/Fe_2_O_3_ increased to 1.4 mA cm^−2^, accompanied by a negative initial potential shift of 37 mV. This shift suggests that the formed g-C_3_N_4_/Fe_2_O_3_ heterojunction promoted rapid charge transfer. Moreover, after anchoring CoSAs on g-C_3_N_4_, CoSAs–g-C_3_N_4_/Fe_2_O_3_ exhibited a photocurrent density of 1.93 mA cm^−2^. This indicates that CoSAs provided additional active sites for water oxidation, which effectively inhibited surface charge recombination and promoted interfacial charge transfer. Compared with g-C_3_N_4_/Fe_2_O_3_, the initial potential of CoSAs–g-C_3_N_4_/Fe_2_O_3_ (0.58 V_RHE_) decreased by 158 mV, indicating a significant contribution from dispersed CoSAs to the acceleration of oxygen evolution reaction (OER) kinetics. This finding was consistent with transient photocurrent test results (Fig. 5b). α-Fe_2_O_3_ exhibited a large transient photocurrent spike owing to the significant occurrence of charge recombination in the bulk and surface of the material.^33,34^ The coupling of α-Fe_2_O_3_ with g-C_3_N_4_, significantly suppressed the transient peak. Additionally, the gradual increase in the photocurrent density of g-C_3_N_4_/Fe_2_O_3_ can be attributed to the built-in electric field generated by the g-C_3_N_4_/Fe_2_O_3_ heterojunction, which facilitated the directional separation of photogenerated charges.^1,35,36^ Compared with g-C_3_N_4_/Fe_2_O_3_, CoSAs–g-C_3_N_4_/Fe_2_O_3_ exhibited a smaller transient spike, which almost disappeared, while the photocurrent further increased. This phenomenon indicates that the dispersed CoSAs cocatalyst promoted sluggish water oxidation kinetics, thereby significantly facilitating charge transfer and separation. Furthermore, the incident photon-to-current efficiency (IPCE) of the sample was calculated to evaluate its solar energy conversion efficiency. At a wavelength (*λ*) of 350 nm, α-Fe_2_O_3_, g-C_3_N_4_/Fe_2_O_3_, and CoSAs–g-C_3_N_4_/Fe_2_O_3_ exhibited IPCE values of 14.5, 26.8, and 40.9%, respectively, indicating a gradually increasing trend (Fig. 5c). Regarding the influence of light collection efficiency, charge separation efficiency, and injection efficiency on IPCE, the IPCE result strongly confirmed the synergistic effect of g-C_3_N_4_ and CoSAs in enhancing the light absorption, promoting charge separation, and improving hole injection efficiency of the pure α-Fe_2_O_3_ photoanode. The applied bias photon-to-current conversion efficiency (ABPE) results exhibited a similar trend (Fig. 5d). The α-Fe_2_O_3_ photoanode exhibited an ABPE value of 0.05% at 1.06 V_RHE_, which was significantly lower than those of g-C_3_N_4_/Fe_2_O_3_ (0.1% at 1.05 V_RHE_) and CoSAs–g-C_3_N_4_/Fe_2_O_3_ (0.18% at 1.03 V_REH_). The increase in ABPE values and the negative shift of the peak position indicated that the introduction of g-C_3_N_4_ and CoSAs can enable efficient charge separation in the low potential range, facilitating better energy conversion.^1,14,22^

**Fig. 6:**
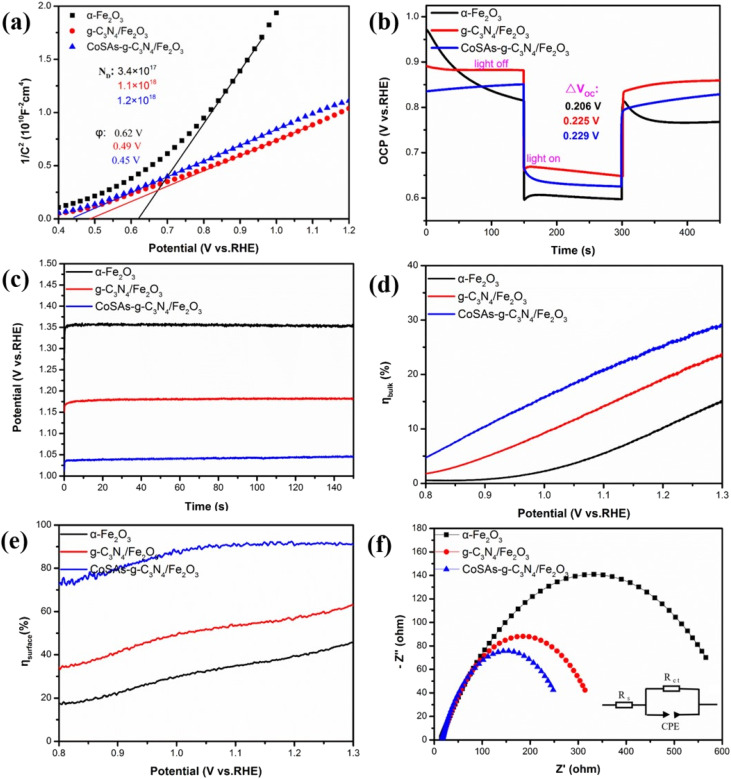
(a) M–S plots; (b) OCP transient decay curves; (c) potential (V_RHE_) *versus* time for the photoelectrodes at 1 mA cm^−2^; (d) *η*_bulk_; (e) *η*_surface_; (f) EIS of α-Fe_2_O_3_, g-C_3_N_4_/Fe_2_O_3_, CoSAs–g-C_3_N_4_/Fe_2_O_3_ photoanodes.

The Mott–Schottky (M–S) plots, open circuit photovoltage (OCP) curves, and the potential required to generate a photocurrent density of 1 mA cm^−2^ were used to further investigate the influence of g-C_3_N_4_ and Co SAs on the PEC performance of α-Fe_2_O_3_. All photoanodes exhibited positive slopes, reflecting their n-type semiconductor characteristics (Fig. 6a).^29,37^ The α-Fe_2_O_3_, g-C_3_N_4_/Fe_2_O_3_, and CoSAs–g-C_3_N_4_/Fe_2_O_3_ photoelectrodes featured carrier densities of 3.4 × 10^17^, 1.1 × 10^18^, and 1.2 × 10^18^ cm^−3^, respectively. The increase in carrier concentration can be attributed to the surface modifications introduced by g-C_3_N_4_ as a heterojunction and CoSAs as a cocatalyst. These modifications significantly inhibited the recombination process of the bulk and surface charge carriers, facilitating the easier release and injection of more photogenerated holes into the electrolyte. Consequently, this led to improved charge separation efficiency and water oxidation performance. Additionally, the successive negative shift in the flat potential of α-Fe_2_O_3_ (0.62 V_RHE_), g-C_3_N_4_/Fe_2_O_3_ (0.49 V_RHE_), and CoSAs–g-C_3_N_4_/Fe_2_O_3_ (0.45 V_RHE_) indicated increased photovoltage, enhanced charge mobility, and improved water oxidation kinetics. Generally, a higher the Δ*V*_OC_ (Δ*V*_OC_ = OCP_dark_ − OCP_light_) indicates a stronger inherent electric field and a greater driving force for carrier separation.^14,38^ The α-Fe_2_O_3_, g-C_3_N_4_/Fe_2_O_3_, and CoSAs–g-C_3_N_4_/Fe_2_O_3_ photoanodes exhibited Δ*V*_OC_ values of 0.206, 0.225, and 0.229 V_RHE_, respectively (Fig. 6b). The g-C_3_N_4_/Fe_2_O_3_ composite exhibited a higher Δ*V*_OC_ than α-Fe_2_O_3_, indicating the formation of a strong built-in electric field resulting from the coupling of g-C_3_N_4_ and α-Fe_2_O_3_. This increased band bending provided an additional driving force for charge separation and effectively suppressed carrier recombination.^39,40^ Upon the introduction of CoSAs, the Δ*V*_OC_ of CoSAs–g-C_3_N_4_/Fe_2_O_3_ further increased, indicating that CoSAs can further reduce carrier recombination and promote charge separation. The potentials required for α-Fe_2_O_3_, g-C_3_N_4_/Fe_2_O_3_, and CoSAs–g-C_3_N_4_/Fe_2_O_3_ to generate a photocurrent density of 1 mA cm^−2^ were 1.36, 1.17, and 1.03 V_RHE_, respectively (Fig. 6c). The significant decrease in overpotential indicated that the synergistic effect of the g-C_3_N_4_/Fe_2_O_3_ heterojunction and CoSAs can improve charge separation and injection efficiency, thereby significantly enhancing PEC water oxidation performance. To accurately assess the effect of g-C_3_N_4_ and CoSAs on the PEC properties of α-Fe_2_O_3_, the bulk charge separation efficiency (*η*_bulk_) and surface charge separation efficiency (*η*_surface_) were independently calculated. This calculation was performed using the LSV data and the integrated photocurrent density (*J*_abs_) (Fig. S7†) with Na_2_SO_3_ as the sacrificial agent. The *η*_bulk_ of pure α-Fe_2_O_3_ considerably increased from 11.5% to 20.6% with the introduction of g-C_3_N_4_, and further increased to 26.3% with CoSAs (Fig. 6d). Notably, both the incorporation of g-C_3_N_4_ as a heterojunction and the introduction of CoSAs as a cocatalyst can reduce the recombination of bulk photogenerated electron–hole pairs and promote charge separation, with the construction of heterojunctions playing a more critical role. Additionally, the *η*_surface_ of α-Fe_2_O_3_ significantly increased from 40.3% to 58.8% with the introduction of g-C_3_N_4_ and further to 91.3% with the addition CoSAs. The highest charge injection efficiency achieved through the introduction of CoSAs can be attributed to the dispersed CoSAs, which effectively extracted holes from the surface of g-C_3_N_4_/Fe_2_O_3_ and provided more active sites to deoxidize water (Fig. 6e).^41^ Electrochemical impedance spectroscopy (EIS) was used to examine the charge transfer resistance at the photoanode interface. All Nyquist plots were fitted using the equivalent circuit (inset of Fig. 6f). The relevant fitting results are summarized in Table S2.† The g-C_3_N_4_/Fe_2_O_3_ composite (337.2 Ω) exhibited a significantly lower charge transfer resistance than α-Fe_2_O_3_ (634.3 Ω), indicating the heterojunction interface facilitated rapid interfacial charge transfer (Fig. 6f). With the anchoring of CoSA cocatalysts, CoSAs–g-C_3_N_4_/Fe_2_O_3_ exhibited the lowest resistance (269.9 Ω), signifying the role of the CoSA cocatalyst in improving the efficiency of surface charge transfer and hole injection. All these results confirm that the synergistic effect of CoSAs and heterojunction fabrication can significantly improve the overall carrier density, charge separation, and injection efficiency, leading to a significant enhancement in PEC water splitting performance.

**Fig. 7:**
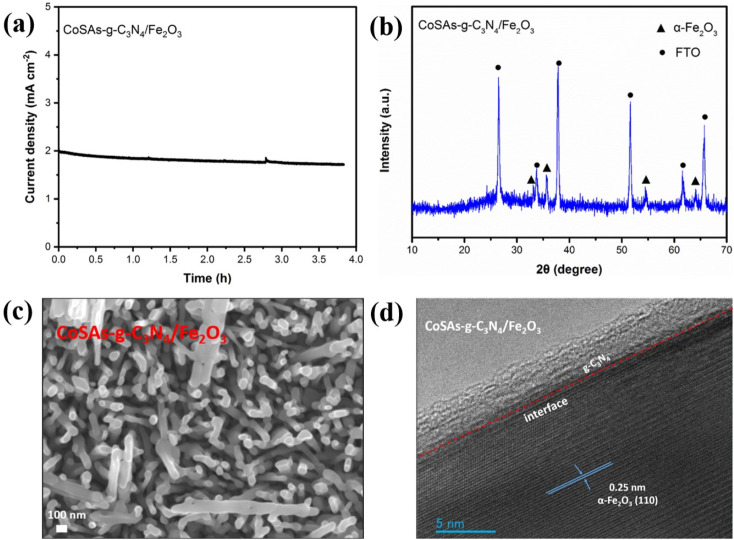
(a) Photocurrent density stability of CoSAs–g-C_3_N_4_/Fe_2_O_3_ photoanodes at 1.23 V_RHE_; (b) XRD pattern; (c) SEM; (d) HRTEM of CoSAs–g-C_3_N_4_/Fe_2_O_3_ photoanodes after long-term stability tests.

Stability is a vital parameter for evaluating the performance of photoelectrodes. Therefore, we investigated the water oxidation stability of CoSAs–g-C_3_N_4_/Fe_2_O_3_, and the relevant results are shown in Fig. 7. After continuous irradiation for 3.8 h (Fig. 7a), the photoanode exhibited good stability and acceptable attenuation of photocurrent density. The XRD (Fig. 7b), SEM (Fig. 7c), and HRTEM (Fig. 7d) images of the photoanodes remained consistent with those obtained before testing. This indicates that the crystal structure and array morphology of the surface CoSAs–g-C_3_N_4_/Fe_2_O_3_ electrode remained unchanged.

**Fig. 8:**
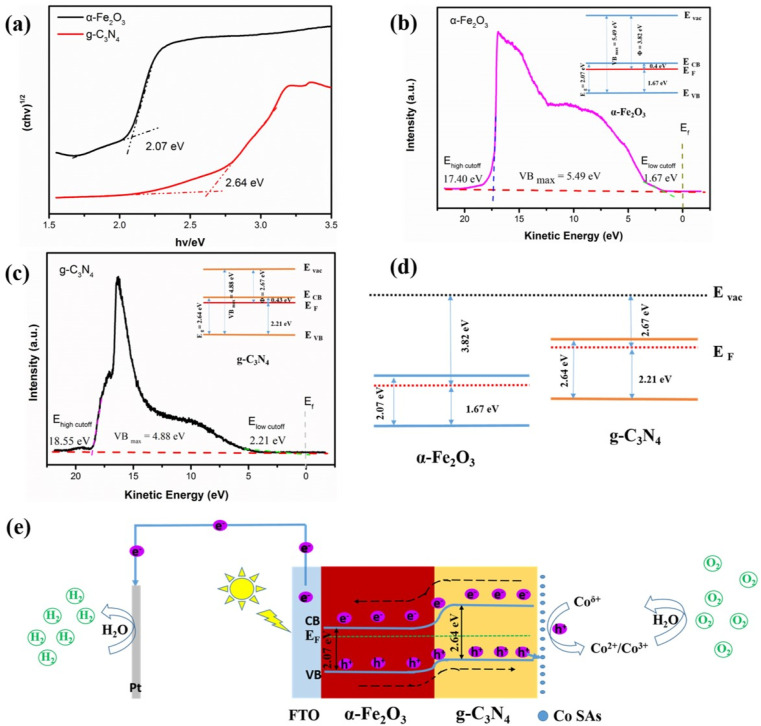
(a) Calculated band gap values (based on UV-Vis absorption spectra) of α-Fe_2_O_3_ and g-C_3_N_4_. Ultra UV photoelectron spectroscopy (UPS) and work function of (b) α-Fe_2_O_3_ and (c) g-C_3_N_4_; (d) energy band diagram of the g-C_3_N_4_/Fe_2_O_3_ heterojunction; (e) Schematic illustration of electron–hole separation of CoSAs–g-C_3_N_4_/Fe_2_O_3_ photoanode for PEC water splitting.

To elucidate possible charge transfer pathways and water oxidation mechanisms, the band positions of α-Fe_2_O_3_ and g-C_3_N_4_ were accurately examined. The energy band location of CoSAs–g-C_3_N_4_/Fe_2_O_3_ was calculated and plotted based on the results of UV-Vis spectroscopy (Fig. S8†) and UPS. The α-Fe_2_O_3_ and g-C_3_N_4_ samples exhibited band gap values of 2.07 and 2.64 eV, respectively (Fig. 8a). The UPS results of the α-Fe_2_O_3_ and g-C_3_N_4_ samples are illustrated in Fig. 8b and c. The work function (*Φ*) indicated the energy of the Fermi level regarding the vacuum level, while |*E*_VBM_| was calculated using eqn (S5) and (S6).† The α-Fe_2_O_3_ and g-C_3_N_4_ samples featured *Φ* values of 3.82, and 2.67 eV, respectively. Additionally, α-Fe_2_O_3_ and g-C_3_N_4_ exhibited |*E*_VBM_| values of 5.49 and 4.88 eV, respectively. Moreover, the inset in Fig. 8b and c shows the band alignment diagrams derived from α-Fe_2_O_3_ and g-C_3_N_4_, respectively. With the use of the band arrangement diagrams obtained from α-Fe_2_O_3_ and g-C_3_N_4_, Fig. 8d illustrates the schematic of the CoSAs–g-C_3_N_4_/Fe_2_O_3_ band location indicating the formation of a typical type-II heterojunction between α-Fe_2_O_3_ and g-C_3_N_4_. Therefore, the potential water oxidation mechanism of CoSAs–g-C_3_N_4_/Fe_2_O_3_ is illustrated in Fig. 8e. Upon contact between α-Fe_2_O_3_ and g-C_3_N_4_, band bending occurred, thereby establishing the Fermi level equilibrium, and forming heterojunction. Upon illumination, the photoelectrons of α-Fe_2_O_3_ and g-C_3_N_4_ were excited from the valence band (VB) to the conduction band (CB), respectively. In the presence of the built-in electric field formed at the interface between α-Fe_2_O_3_ and g-C_3_N_4_, photogenerated charges underwent rapid separation and transfer, effectively inhibiting electron–hole pairs recombination. Photoelectrons transitioned from the CB of g-C_3_N_4_ to the CB of α-Fe_2_O_3_ and then traveled through the external circuit to the Pt electrode, facilitating the reduction of water to produce hydrogen. Furthermore, the VB holes in α-Fe_2_O_3_ rapidly migrated to the VB of g-C_3_N_4_, where they were further captured by the highly dispersed CoSAs. Owing to the strong oxidation of these photogenerated holes, Co^*δ*+^ can be oxidized to Co^2+^/Co^3+^, which oxidized water to produce oxygen. Moreover, the reduction of Co^2+^/Co^3+^ ions to Co^*δ*+^, enabled the efficient separation of photogenerated electrons and holes.

## Conclusions

We successfully prepared a novel CoSAs–g-C_3_N_4_/Fe_2_O_3_ photoanode with excellent PEC performance by coupling g-C_3_N_4_ with α-Fe_2_O_3_ and anchoring CoSAs on g-C_3_N_4_. Compared with pure α-Fe_2_O_3_, the g-C_3_N_4_/Fe_2_O_3_ heterostructure significantly inhibited the recombination of photogenerated charges. The incorporation of CoSAs into the photoanode structure further improved the charge separation efficiency and injection efficiency of α-Fe_2_O_3_, thereby promoting reaction kinetics. The synergistic effect of Co SAs and g-C_3_N_4_ contributed to the excellent PEC performance of CoSAs–g-C_3_N_4_/Fe_2_O_3_. The optimized CoSAs–g-C_3_N_4_/Fe_2_O_3_ photoanode exhibited a maximum photocurrent density of 1.93 mAcm^−2^ at 1.23 V_RHE_, which was 3.22 times that of pure α-Fe_2_O_3_, with a negative shift in initial potential by 195 mV. This study provides a promising approach for developing efficient and stable single atom photoanodes for PEC water splitting applications.

## Data availability

All experimental details and characterisation data can be found in the ESI.†

## Author contributions

J. W., L. J. and W. L. designed the experiment and wrote the manuscript. J. W., B. H., H. D. and N. W. conducted the experiments. J. W., X. D., M. L. and H. C. analysed data. All authors discussed the results at all stages and participated in the development of the manuscript.

## Conflicts of interest

The authors declare no conflict of interest.

## Supplementary Material

SC-015-D4SC03442B-s001
