# Supplementary material for: Enhanced photoelectrochemical water splitting performance of α-Fe2O3 photoanodes through Co-modification with Co single atoms and g-C3N4
Source: Chem Sci. 2024 Jul 5;15(32):12973–82. doi: 10.1039/d4sc03442b (PMC11323335; doi:10.1039/d4sc03442b)
Supplement: SC-015-D4SC03442B-s001 [file SC-015-D4SC03442B-s001.pdf]

Electronic Supplementary Material (ESI) for Chemical Science.
This journal is © The Royal Society of Chemistry 2024

**Supporting information**

**Enhanced Photoelectrochemical Water Splitting Performance of α-$Fe_2O_3$ Photoanodes through Co-modification with Co Single Atoms and g-$C_3N_4$**

Juan Wu[a], Xiaodi Du[b], Mingjie Li[c], Hongyu Chen[a], Bin Hu[a], Hongwei Ding[a], Nannan Wang[a], Lin Jin[a]*and Weisheng Liu[d]*

[a]Henan Key Laboratory of Rare Earth Functional Materials, International Joint Research Laboratory for Biomedical Nanomaterials of Henan, Zhoukou Normal University, Zhoukou 466001, P. R. China.

[b]College of Chemistry and Chemical Engineering, Zhoukou Normal University, Zhoukou 466001, P. R. China.

[c]Library, Zhoukou Normal University, Zhoukou 466001, P. R. China.

[d]Key Laboratory of Nonferrous Metal Chemistry and Resources Utilization of Gansu Province and State Key Laboratory of Applied Organic Chemistry, College of Chemistry and Chemical Engineering, Lanzhou University, Lanzhou 730000, P. R. China.

E-mail: jinlin_1982@126.com
E-mail: liuws@lzu.edu.cn.

## 1. Experimental section

### 1.1. Materials

Ferric chloride ($FeCl_3 \cdot 6H_2O$, 99%), urea ($CO(NH_2)_2$) and ethylene glycol ($C_2H_6O_2$) were purchased from Shanghai Aladdin Biochemical Technology Co., Ltd. Cobalt nitrate hexahydrate ($Co(NO_3)_2 \cdot 6H_2O$, 99%), melamine ($C_3N_3(NH_2)_3$, 99%), potassium hydroxide (KOH, 95%) were purchased from Beijing Yinuokai Technology Co., Ltd. sodium sulfite ($Na_2SO_3$) was purchased from Sinopharm Chemical Reagent Co., Ltd. All reagents were used directly without further purification. The fluorine-doped tin oxide (FTO) glass (1cm×4 cm) were purchased from Foshan Yuanjingmei Glass Co., Ltd. which were ultrasonically cleaned in deionized water, ethanol, acetone, ethanol and deionized water for 10 minute before use.

### 1.2 Characterizations:

All the samples were characterized by Field emission scanning electron microscopy (FESEM, JEOL JSM-IT800), TEM (Hitachi-H-7650), and atomic-resolution TEM with spherical aberration (Cs) corrector (CS-FE-TEM, JEM-ARM200F), X-ray diffraction spectra (XRD, Bruker D8-Discove), X-ray photoelectron microscope (XPS, Thermo SCIENTIFIC Nexsa), UV-vis absorbance spectrometer (UV-vis, Shimadzu UV-2600). The photoluminescence (PL) spectra were determined with light at an excitation wavelength of 415 nm at ambient temperature. The work functions and valence bands were determined by Ultraviolet photoelectron spectroscopy (UPS; conducted on a Kratos Axis Ultra DLD, He I

photon source of 21.2 eV). Co K-edge analysis was performed with Si (111) crystal monochromators at the BL11B beamlines at the Shanghai Synchrotron Radiation Facility (SSRF) (Shanghai, China). Before the analysis at the beamline, samples were pressed into thin sheets with 1 cm in diameter and sealed using Kapton tape film. The XAFS spectra were recorded at room temperature using a 4-channel Silicon Drift Detector (SDD) Bruker 5040. Co K-edge extended X-ray absorption fine structure (EXAFS) spectra were recorded in fluorescence mode. The spectra were processed and analyzed by the software codes Athena and Artemis.

**1.3 Photoelectrochemical (PEC) measurements:**

All photoelectrochemical experiments were performed on a CHI 660E electrochemical workstation (Chenhua Instrument Company, Shanghai, China). The obtained photoanodes were used as the working electrodes, saturated Ag/AgCl was used as the reference electrode, and a platinum network was used as the counter electrode. 1 M KOH solution (pH = 13.6) was used as the electrolyte (except for measuring the surface charge transfer efficiency). A 300 W xenon lamp with an AM 1.5G filter was used to simulate sunlight (100 mW $cm^{-2}$). All test voltages vs Ag/AgCl were converted to the reversible hydrogen electrode (RHE) through the formula $E_{RHE} = E_{Ag/AgCl} + E°_{Ag/AgCl} + 0.059$ pH, where $E_{Ag/AgCl}$ is the measured potential and $E°_{Ag/AgCl} = 0.197$ V vs the normal hydrogen electrode (NHE) at 25°C. The I-V characteristics of the working electrodes were measured by sweeping the potential from -0.6 to +0.6 V vs the Ag/AgCl reference electrode at a scan rate of 10 mV $s^{-1}$. Electrochemical impedance spectroscopy (EIS) was performed at +0.05

V vs Ag/AgCl with an alternating current voltage of 10 mV, and the frequency range was fixed from 0.1 Hz to 100 kHz under irradiation of AM 1.5G. The incident photon to current efficiency (IPCE) was measured through a full solar simulator (Newport, Model 9600, 300W Xe arc lamp) and a motorized monochromator (Oriel Cornerstone 130 1/8 m).

### 1.4. Calculation equations

The incident photon-to-current conversion efficiency (IPCE) were obtained at 1.23 V vs. RHE with the wavelength range 300-650 nm. The IPCE is calculated by the following equation:

$$\mathrm{IPCE} = \frac{1240 \times J(mA/cm^2)}{P_{light}(mW/cm^2) \times \lambda(nm)} \qquad \text{(S1)}$$

Where λ is the test-wavelength, J and $P_{light}$ are the measured photocurrent density and light power density at some specific wavelength.

Applied bias photon-to-current efficiency (ABPE) can be calculated using the following equation:

$$\mathrm{ABPE}\ (\%) = \frac{J \times (1.23 - V_b)}{P_{light}} \times 100\% \qquad \text{(S2)}$$

J refers to the photocurrent density (mA $m^{-2}$) obtained from the electrochemical work station. $V_b$ is the applied bias vs. RHE (V), and $P_{light}$ is the total light intensity of AM 1.5 G (100 mW $cm^{-2}$).

The surface separation efficiency ($\eta_{surface}$) of the photoanode can be calculated by the following formula:

$$\eta_{surface} = \frac{J_{H_2O}}{J_{Na_2SO_3}} \qquad \text{(S3)}$$

Where $J_{H_2O}$ and $J_{Na_2SO_3}$ are the photocurrent densities obtained in 1 M KOH electrolyte and 1M $Na_2SO_3$ electrolyte (pH = 9.5).

Mott-Schottky analysis was conducted with the DC potential scanning from – 0.6 to + 0.2V vs. Ag/AgCl at a frequency of 1 kHz in the dark. The carrier density can be measured as the following formula:

$$Nd = \frac{2}{e_0 \varepsilon \varepsilon_0}\left[\frac{d\left({}^{1}/_{C^2}\right)}{dv}\right]^{-1} \qquad (S4)$$

Where $\varepsilon_0$, $\varepsilon$, $e_0$ represent the permittivity of free space ($8.86\times10^{-12}$ F $m^{-2}$), the dielectric constant of $Fe_2O_3$ (80), $e_0$ is electron charge ($1.6\times10^{-19}$ C), respectively. C (F $cm^{-2}$) is the space charge capacitance in the semiconductor (obtained from M-S curves), V is the applied potential for M-S curves.

$$\Phi = h\nu - E_{\text{high cutoff}}, \ (h\nu \approx 21.22 \text{ eV}) \qquad (S5)$$

$$|E_{VBM}| = h\nu - (E_{\text{high cutoff}} - E_{\text{low cutoff}}) \qquad (S6)$$

## Figures

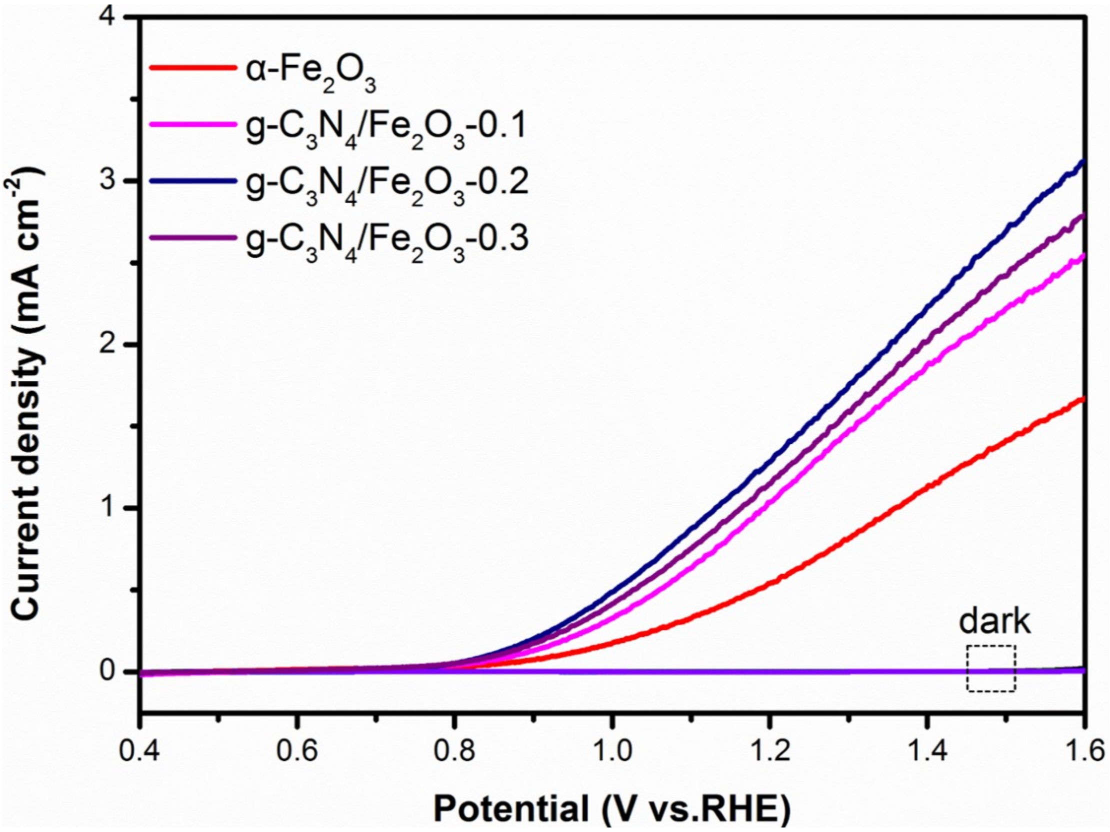


Fig. S1 LSV curves of α-$Fe_2O_3$, g-$C_3N_4$/$Fe_2O_3$-0.1, g-$C_3N_4$/$Fe_2O_3$-0.2, and g-$C_3N_4$/$Fe_2O_3$-0.3.

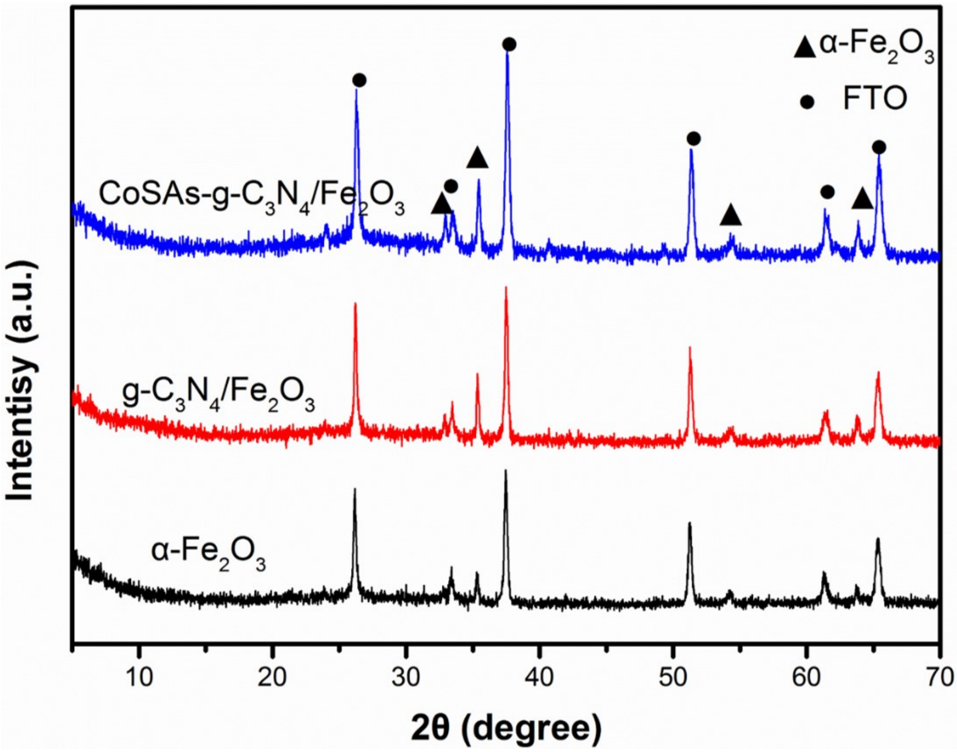


Fig. S2 XRD patterns of α-$Fe_2O_3$, g-$C_3N_4$/$Fe_2O_3$ and CoSAs-g-$C_3N_4$/$Fe_2O_3$.

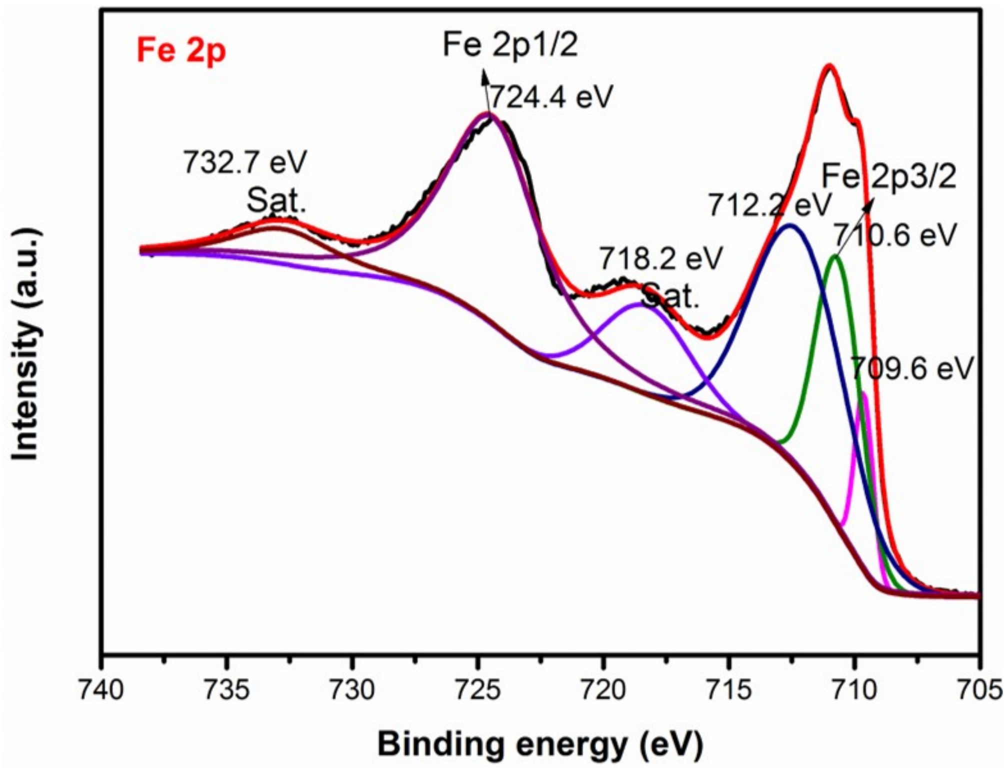


Fig. S3 XPS spectra of Fe 2p for CoSAs-g-$C_3N_4$/$Fe_2O_3$.

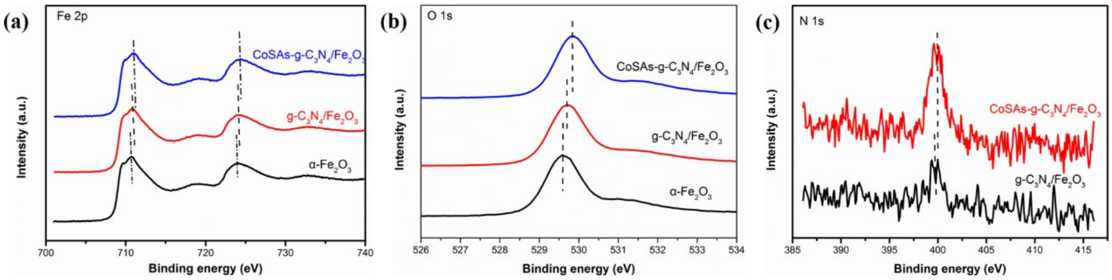


Fig. S4 (a, b) High-resolution XPS spectra of Fe 2p, O1s, respectively, for the α-$Fe_2O_3$, g-$C_3N_4$/ $Fe_2O_3$ and CoSAs-g-$C_3N_4$/$Fe_2O_3$. (c) High-resolution XPS spectra of N 1s for the g-$C_3N_4$/ $Fe_2O_3$ and CoSAs-g-$C_3N_4$/$Fe_2O_3$.

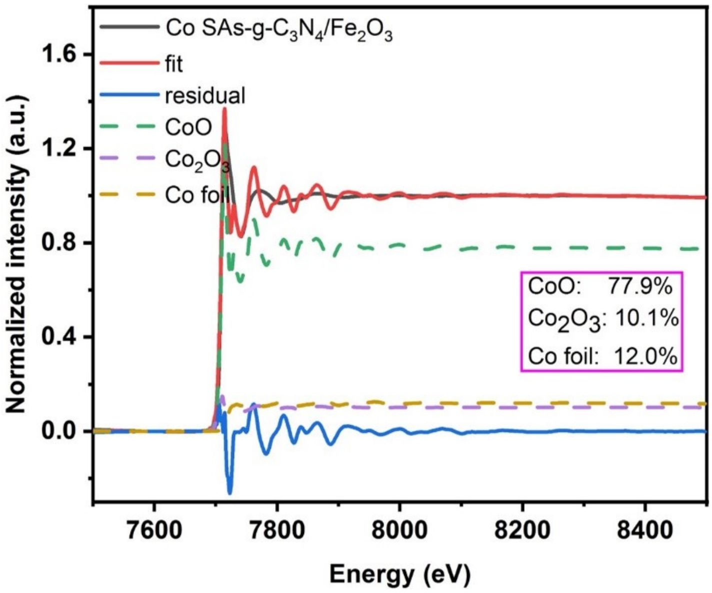


Fig. S5 The linear combination fitting result of the Co SAs-g-$C_3N_4$/$Fe_2O_3$.

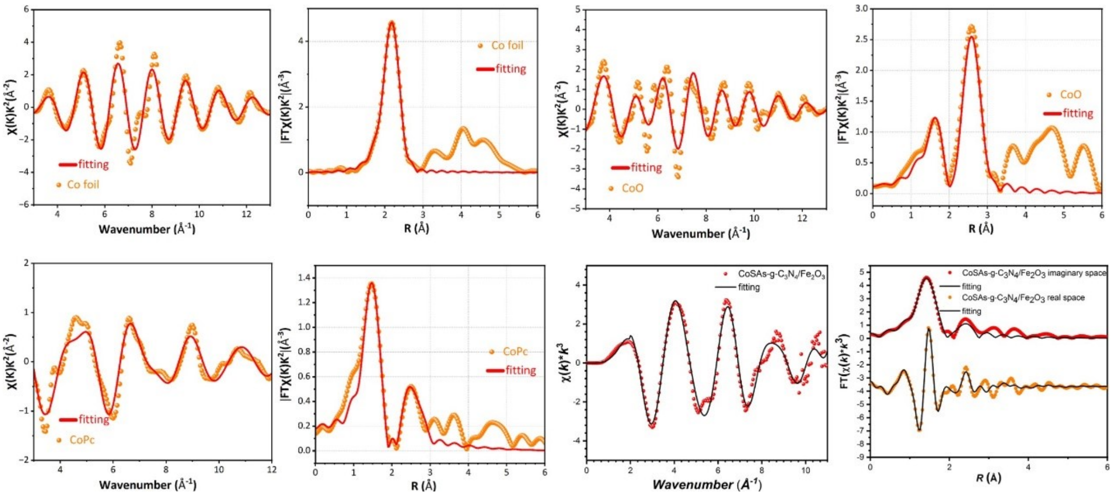


Fig. S6 EXAFS R-space and K-space fitting curve for Co foil, CoO, CoPc and CoSAs-g-$C_3N_4$/$Fe_2O_3$.

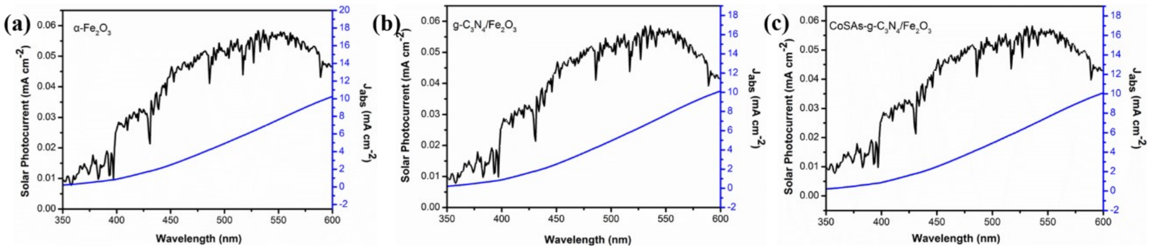


Fig. S7 The calculated current density flux and integrated current density ($J_{abs}$) of (a) α-$Fe_2O_3$, (b) g-$C_3N_4$/$Fe_2O_3$ and CoSAs- g-$C_3N_4$/$Fe_2O_3$.

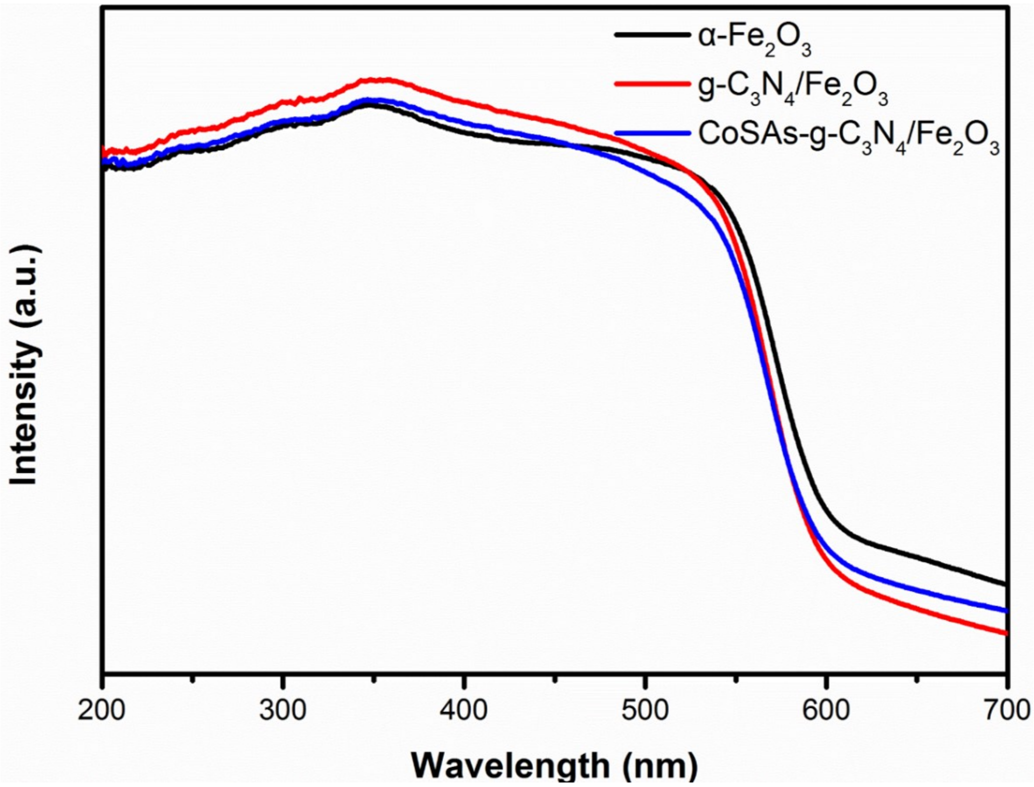


Fig. S8 UV-Vis absorption spectra.

Table S1. EXAFS fitting parameters at the Co K-edge for various samples

| Sample | Shell | $CN^{a}$ | $R$(Å)$^{b}$ | $\sigma^2$(Å$^2$)$^{c}$ | $\Delta E_0$(eV)$^{d}$ | $R$ factor |
|---|---|---|---|---|---|---|
| Co foil | Co-Co | 12.0* | 2.49±0.01 | 0.0062 | 8.4 | 0.0018 |
| CoO | Co-O | 6.0* | 2.12±0.01 | 0.0062 | -0.2 | 0.0197 |
| | Co-Co | 12.0* | 3.00±0.01 | 0.0061 | -3.0 | |
| CoPc | Co–N | 4.4±0.4 | 1.90±0.01 | 0.0015 | 6.3 | 0.0142 |
| | Co-C | 7.7±1.6 | 2.89±0.01 | 0.0189 | -5.8 | |
| Co sample | Co–N | 4.1±0.2 | 1.955±0.0133 | 0.0091±0.0027 | -8.8±0.7 | 0.00171 |
| | Co-C | 1.1±0.4 | 2.872±0.033 | 0.0020 | -8.8±0.7 | |

$^{a}CN$, coordination number; $^{b}R$, distance between absorber and backscatter atoms; $^{c}\sigma^2$, Debye-Waller factor to account for both thermal and structural disorders; $^{d}\Delta E_0$, inner potential correction; $R$ factor indicates the goodness of the fit. $S_0^2$ was fixed to 0.9. A reasonable range of EXAFS fitting parameters: $0.700 < S_0^2 < 1.000$; $CN > 0$; $\sigma^2 \geq 0.002$ Å$^2$; $|\Delta E_0| < 15$ eV; $R$ factor < 0.02 。

Table S2. Fitting data of the EIS on α-$Fe_2O_3$, g-$C_3N_4$/$Fe_2O_3$, and CoSAs-g-$C_3N_4$/$Fe_2O_3$ derived from Figure 6f.

| Sample | Rs (Ω) | Rct (Ω) |
|---|---|---|
| α-$Fe_2O_3$ | 13.41 | 634.3 |
| g-$C_3N_4$/$Fe_2O_3$ | 16.52 | 337.2 |
| CoSAs-g-$C_3N_4$/$Fe_2O_3$ | 16.12 | 269.9 |
